# A multi-centre, UK-based, non-inferiority randomised controlled trial of 4 follow-up assessment methods in stroke survivors

**DOI:** 10.1186/s12916-019-1350-5

**Published:** 2019-07-02

**Authors:** Jonathan Hewitt, Anna Pennington, Alexander Smith, Stephanie Gething, Michelle Price, James White, Richard Dewar, Ben Carter

**Affiliations:** 1 Division of Population Medicine, School of Medicine, Cardiff University, Cardiff, UK; 2 Aneurin Bevan University Health Board, Newport, South Wales UK; 3 Ynys Y Plant, Plantation Lane, Newtown, SY16 1LH UK; 4 Prince Charles Hospital, Cwm Taf NHS Trust, Prince Charles Hospital, Merthyr Tydfil, CF47 9DT UK; 5 Department of Biostatistics and Health Informatics, Institute of Psychiatry, Psychology & Neuroscience, King’s College London, London, UK

**Keywords:** Stroke, Follow-up method, Online assessment, Non-inferiority

## Abstract

**Background:**

Recovery following a stroke is a long and ongoing process. Post-stroke follow-up after leaving the hospital is recommended. Methods for follow-up patients include face-to-face, via the telephone, post or online (internet). However, there is a debate which method is preferred by patients. This study aimed to determine whether telephone interview, online questionnaire and postal questionnaire were as acceptable as face-to-face follow-up.

**Methods:**

In a blinded, UK-wide, multi-centre, Zelen’s designed, 4-arm (postal, online, telephone, compared to face-to-face), pragmatic non-inferiority randomised controlled trial of the mode of administration, stroke survivors were randomised to postal, online, telephone and face-to-face assessment, in an equal ratio (1:1:1:1). The primary outcome was the proportion of participants that responded to the three allocation groups, compared to the face-to-face group. Subgroup analyses for age, aphasia and type and severity of stroke were carried out. A non-inferiority margin of 0.025 was used, and Holm-Bonferroni multiplicity adjustment was made.

**Results:**

Of the 2074 eligible patients randomised, 55% were male (1142/2074), with an average age of 73.0 years old (SD = 13.2). Of those randomised, 22% (116/525), 9% (47/515) and 20% (101/513) responded in postal, online and telephone, respectively, compared to 17% (89/521) in the face-to-face group. The reduction in the online response rate compared to face-to-face was found to be both inferior and not non-inferior and estimated as an 8% reduction (95% CI 3.9 to 12.0%; *p* < 0.001). The association with lower online completion was present regardless of age, stroke type (haemorrhage or infarct) and stroke severity. In haemorrhagic stroke, the reduction in response online, compared to face-to-face, was 21% (95% CI 10 to 32%; *p* value = 0.002). A secondary analysis found non-aphasic stroke survivors preferred postal completion adjusted odds ratio of 1.43 (95% CI 1.04 to 1.95; *p* = 0.026).

**Conclusions:**

The study found that fewer stroke survivors completed follow-up assessment using an online method, compared to face-to-face. This finding was present in all age groups. Caution should be employed when considering online follow-up methods in stroke survivors, particularly in those who have experienced a cerebrovascular haemorrhage.

**Trials registration:**

ClinicalTrials.gov, NCT03177161. Registered on 6 June 2017.

**Electronic supplementary material:**

The online version of this article (10.1186/s12916-019-1350-5) contains supplementary material, which is available to authorized users.

## Background

Clinical guidelines recommend long-term follow-up after stroke to improve a range of important clinical outcomes [1]. Follow-up assessment can take arrange of forms, but many are increasingly being conducted using an online format. There are no randomised controlled trials which consider the best method of delivering post-stroke follow-up assessment.

Using a 15-question stroke-specific patient-reported outcome measure [2, 3], this research study aimed to provide an evidence base for the optimal method of follow-up in stroke survivors. The study evaluated online, postal and telephone modes of follow-up compared to a face-to-face assessment. This was achieved utilising a pragmatic study design offering UK-wide generalisable results of the acceptability of the methods under investigation, to inform clinicians and commissioners of stroke services of the best methods of delivering follow-up assessment following a stroke.

## Methods

### Trial design

The full trial protocol has been published [4], but in brief, the study applied Zelen’s design [5] across multiple stroke units in England and Wales. This was a UK-wide, multi-centre, 4-arm (postal, online, telephone, compared to face-to-face), pragmatic non-inferiority randomised controlled trial of the mode of administration of 15 patient-reported questions specifically for stroke survivors, conducted at the 6-month post-stroke follow-up. The questionnaire can be seen in Additional file 1: Figure S1.

Inclusion criteria included adult participants, with a clinical diagnosis of stroke, both ischaemic or haemorrhagic. Exclusion criteria were patients with a clinical diagnosis of transient ischaemic attack, subarachnoid haemorrhage or stroke survivors undergoing end of life care.

The potentially eligible participants received an invitation letter, a participant information sheet (PIS) and a consent form via the post. The PIS invited the participant to consent to the study and receive their 6-month review and the 15 questions via one of the four methods to which they had been allocated to. A proxy consent option was also included. Participants who did not respond were invited a second time, 2 weeks later; if no further response was received, they were deemed not to be interested in taking part in the study and not contacted further.

The randomisation sequence was generated with a random varying permuted block design (block sizes of 4 to 16) to four methods of assessment in equal allocation (1:1:1:1), stratified by the hospital. The allocation sequence was concealed from those involved with the recruitment, the chief investigator and statistician. The four different methods of assessment were as follows:

*Face-to-face*: In the UK, all stroke survivors receive a 6-month post-diagnosis review appointment with either a clinical nurse specialist (CNS) in stroke or their clinician, depending on the routine practice within their hospital. Any participant who had consented to the study underwent their 15-question assessment during this visit.

*Telephone interview*: An appointment was sent to the participants to receive a telephone interview for the 6-month review appointment conducted by either a CNS or a clinician depending on local practice.

*Postal questionnaire*: Participants received a postal version of the 15 questions; this included a prepaid and addressed envelope in which to return the questionnaire.

*Online questionnaire*: Participants received a postal invitation to access an online version of the 15 questions via a secure web address. This was in the form of a URL address, which required typing into a web browser for navigation to the questions.

### Outcomes

The primary outcome was the proportion of participants that responded to the three allocation groups (completed/randomised), compared to the face-to-face group, to adjust for the multiplicity of three pair-wise comparisons.

For the secondary outcomes, the primary outcome is re-analysed using a mixed effects logistic regression model to adjust for covariates and confounders.

Baseline demographic data were collected on all patients contacted. Data included age, sex, date of birth, date of diagnosis and district-level postcode. Clinical health-related data, which included individual National Institutes of Health Stroke Score (NIHSS) on admission, classification of stroke (i.e. infarct or haemorrhage using ICD classifications), Modified Rankin Score on discharge, whether the participant received thrombolysis and whether or not the participant had aphasia, were recorded.

### Data analysis and statistics

#### Sample size justification

The sample size is defined in the protocol with a non-inferiority margin of 0.025 [4].

### Description of the population

A baseline descriptive analysis was conducted to confirm the random allocation of participants to the four groups is distributed with approximate balance.

### Data analysis

The primary outcome analysis used a two-sample difference of proportions that compared the three groups separately versus face-to-face completion (as the gold standard). A 95% confidence interval and *p* value were calculated, and using a fixed-margin approach, the difference was compared against the non-inferiority margin (*Δ* = − 0.025) to conclude non-inferiority or against no difference to conclude superiority/inferiority. A Holm-Bonferroni adjustment was made to the interpretation of the *p* value in each case. Stata statistical software was used throughout.

The secondary outcome analysis fitted a mixed effects multivariable logistic regression, with each hospital fitted as random intercept effect, and patient age, sex, NIHHS score on admission and stroke type as fixed effects (as a conditional likelihood).

### Subgroup analysis

We analysed the following subgroups: aphasia status, type of stroke, severity of stroke and patient age.

### Population under investigation and handling of missing data

Due to using Zelen’s design, we anticipated inflated loss to follow-up. All contacted eligible patients were included in the analysis. Our analysis population was a modified intention-to-treat population.

### Blinding of personnel

The chief investigator (did not recruit or follow up patients) and trial statistician were fully blinded to the allocation until the planned unblinding of the trial at the final Trial Steering meeting. King’s College London Clinical Trial Unit (KCTU) standard operating procedures (SOP) were used throughout this study. The definition of being fully blind was no knowledge of any post-baseline outcome data split by arm, prior to database lock. Planned unblinding occurred by the Chair of the Trial Steering Committee at the final study meeting.

### Ethical arrangements

The study was approved by North West - Greater Manchester South Research Ethics Committee via the Health Research Authority (HRA) NHS research ethics committee on 26 April 2017 and sponsored by Aneurin Bevan University Health Board (REC number 17/NW/0269, IRAS reference number 222226).

## Results

This trial recruited from 14 UK centres (Additional file 1: Table S1) between 6 July 2017 and 22 January 2018. There were 2217 participants screened for eligibility, and 143 were excluded from the study because they did not meet the inclusion criteria. In total, 2074 eligible participants were randomised. The consort study flow diagram is provided in Additional file 1: Figure S2. In total, 23% of the population consented to the study, and 18% returned the questionnaire.

Of the 2074 patients randomised, 55% were male (1142/2074), with an average age of 73.0 years old (SD = 13.2). No differences were found in the baseline patient demographics and clinical characteristics for the four allocation groups (Table 1).

**Table 1:** Baseline descriptions for the four allocation groups; the number of participants is shown within each group, with the associated percentage across all four allocation groups

		Allocation group				
		Postal	Online	Face-to-face	Telephone	Total (*n* = 2074)
Total		525 (25%)	515 (25%)	521 (25%)	513 (25%)	
Sex	Female	233 (44%)	238 (46%)	231 (44%)	230 (45%)	932 (45%)
	Male	292 (56%)	277 (54%)	290 (56%)	283 (55%)	1142 (55%)
Age	Standard deviation	73.9 (12.9)	72.6 (13.4)	72.6 (13.4)	72.6 (13.3)	73 (13.2)
Stroke severity	Non-mild	245 (47%)	272 (53%)	265 (51%)	250 (49%)	1032 (50%)
	Moderate to severe	280 (53%)	243 (47%)	256 (49%)	263 (51%)	1042 (50%)
Thrombolysis	No	428 (82%)	422 (82%)	429 (82%)	421 (82%)	1700 (82%)
	Yes	81 (15%)	73 (14%)	73 (14%)	79 (15%)	306 (15%)
	Missing	16 (3%)	20 (4%)	19 (4%)	13 (3%)	68 (3%)
Modified Rankin	0	97 (18%)	93 (18%)	98 (19%)	100 (19%)	388 (19%)
	1	89 (17%)	82 (16%)	97 (19%)	95 (19%)	363 (18%)
	2	55 (10%)	59 (11%)	61 (12%)	60 (12%)	235 (11%)
	3	80 (15%)	84 (16%)	75 (14%)	73 (14%)	312 (15%)
	4	52 (10%)	49 (10%)	55 (11%)	46 (9%)	202 (10%)
	5	34 (6%)	27 (5%)	22 (4%)	26 (5%)	109 (5%)
	Missing	118 (22%)	121 (23%)	113 (22%)	113 (22%)	465 (22%)
Aphasia	No	148 (28%)	147 (29%)	153 (29%)	153 (30%)	601 (29%)
	Yes	53 (10%)	49 (10%)	55 (11%)	46 (9%)	203 (10%)
	Missing	324 (62%)	319 (62%)	313 (62%)	314 (62%)	1297 (63%)
Stroke classification	Cerebral infarct	467 (89%)	466 (90%)	449 (86%)	459 (89%)	1841 (87%)
	Cerebrovascular haemorrhage	50 (10%)	44 (9%)	64 (12%)	46 (9%)	204 (10%)
	Stroke—not specified	7 (1%)	5 (1%)	7 (1%)	8 (2%)	27 (1%)
	Missing	1 (0%)	0 (0%)	1 (0%)	0 (0%)	41 (2%)

### Primary analysis of the primary outcome

#### Primary outcome

Of the 2074 patients randomised, 22% (116/525), 9% (47/515) and 20% (101/513) responded via post, online and telephone, respectively, compared to 17% (89/521) in the face-to-face allocation group.

**Fig. 1:**
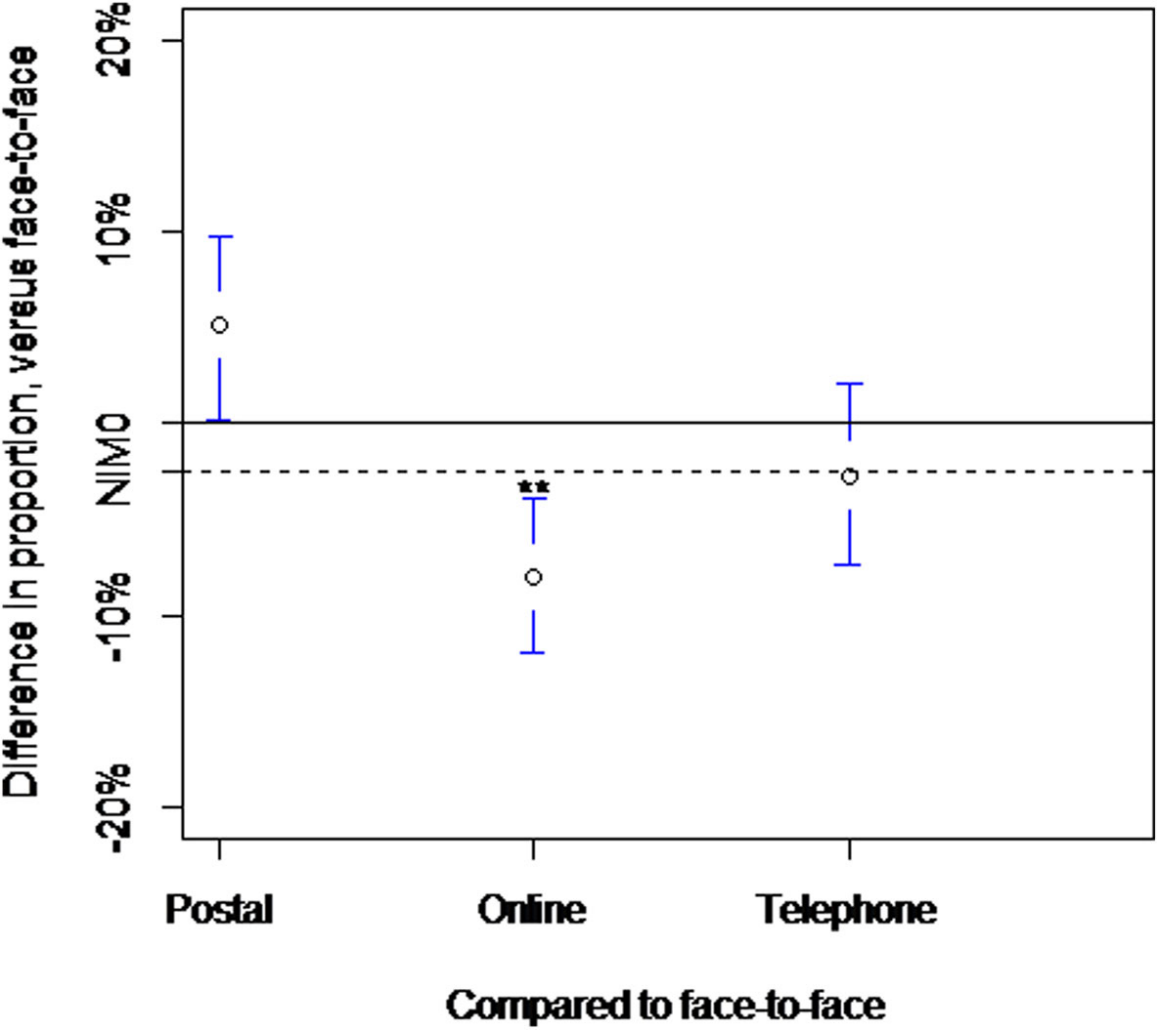
The completion rates for postal, online and telephone follow-up methods, compared to face-to-face are shown with the associated 95% confidence intervals. The solid horizontal line is the line of no difference, and the non-inferiority margin (NIM) is shown with the broken horizontal line

There was very clear evidence that the online group had both an inferior and not non-inferior response rate compared to face-to-face after fitting a non-inferiority margin of 0.025. The reduction in the online response rate compared to face-to-face was estimated as 8% (95% CI 3.9 to 12.0%; *p* < 0.001) (Fig. 1). Postal assessment was found to be not inferior to face-to-face, and there was evidence that postal assessment had an increased response of 5% (95% CI 0.2 to 10%, *p* value = 0.04). However, after applying a Holm-Bonferroni adjustment, this was not statistically significant.

### Secondary outcomes

After adjustment by fitting a mixed effects multivariable logistic model, adjusted for sex, age, site, stroke severity and stroke classification, the findings from the primary analysis were unchanged. The adjusted odds ratio (aOR) was 0.46 (0.31–0.67) in the online group. Thus, there was a reduction in odds of 54% from online compared to face-to-face responses (95% CI 33 to 69%; *p* < 0.001). There was evidence that the response was increased in postal responses by 43% (95% CI 4 to 95%; *p* = 0.026); however, this was not maintained after the Holm-Bonferroni adjustment. No difference was found between the telephone and face-to-face responses.

### Subgroup analyses

#### Patients without aphasia

There were 601 patients that did not have aphasia, 36% (39/109), 10% (15/147) and 15% (23/153) responded to postal, online or telephone, respectively, compared to 15% (23/153) in the face-to-face allocation group. There was evidence that those patients that were not aphasic had an increased response in postal responses with an increased response of 11% (95% CI 2 to 20%; *p* = 0.015). Overall, there was a high proportion of missing data for stroke survivors with aphasia (1270, 61%) (Table 2).

**Table 2:**
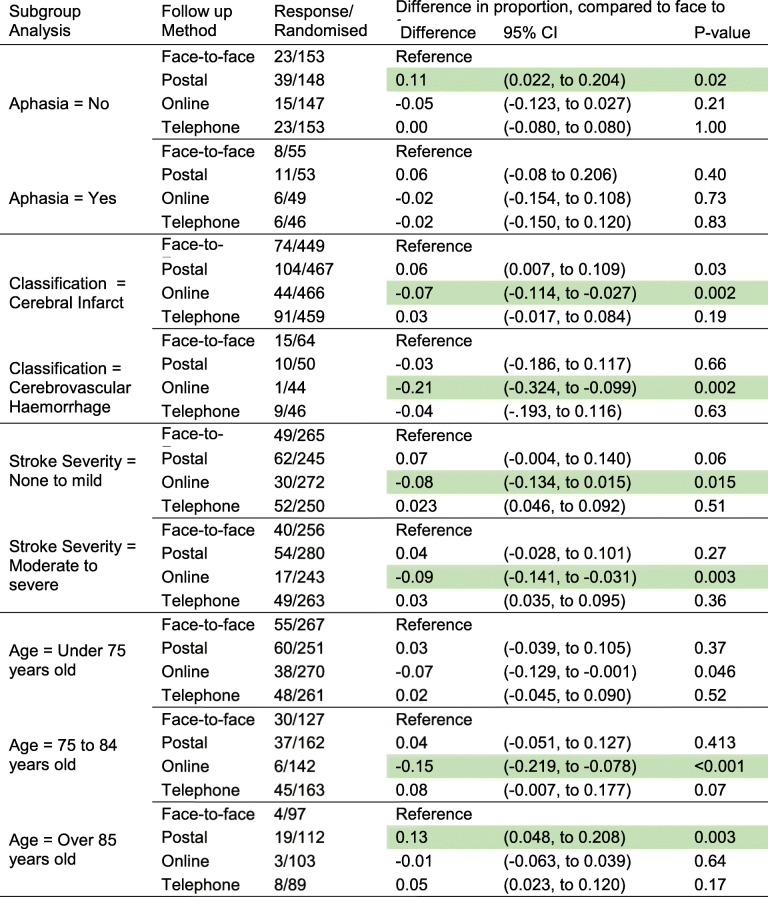
Subgroup analyses. The difference in the proportion in completion rates, compared to face-to-face, with 95% confidence interval and *p* value. Rows in green indicate evidence of a difference in response rate compared with after the 95% adjusted Holm-Bonferroni threshold

#### Patients with aphasia

There were 203 patients recorded as having aphasia, 21% (11/53), 12% (6/49) and 13% (6/46) responded in the postal, online and telephone groups, respectively, compared to 15% (8/55) in the face-to-face group. No differences were found in this subgroup, but this comparison had reduced power due to five sites failing to report these data.

#### Patients with cerebral infarct

There were 1841 participants with a cerebral infarct. Of these, 22% (104/467) responded via post, 9% (44/466) via the online format and 20% (91/459) via the telephone compared to 16% (74/449) in the face-to-face allocation group. There was evidence that patients who experience a cerebral infarct were not followed up online; the reduction was 7% (95% CI 3 to 11%; *p* = 0.002). There was an increase in the response in the postal group, compared to face-to-face of 16% (95% CI 1 to 11%; *p* = 0.03).

#### Patients with cerebrovascular haemorrhage

There were 204 patients with a cerebrovascular haemorrhage, 20% (10/50), 2% (1/44) and 20% (9/46) responded in postal, online and telephone, respectively, compared to those allocated to 23% (15/64) face-to-face. There was evidence of a reduction in response in online, compared to face-to-face, with a reduction of 21% (95% CI 10 to 32%; *p* value = 0.002).

#### Patients with no greater than mild stroke severity

There were 1032 patients with no or mild symptoms, 25% (62/245), 11% (30/272) and 21% (52/250) responded allocated to postal, online and telephone, respectively, compared to 18% (49/265) in the face-to-face group. There was evidence that there was a reduction in response in online of 8% (95% CI 2 to 13%; *p* = 0.015).

#### Patients with moderate to severe stroke severity

There were 1042 patients with moderate and severe symptoms, 19% (54/280), 7% (17/243) and 19% (49/263) responded allocated to postal, online and telephone, respectively, compared to 16% (40/256) in the face-to-face group. There was evidence that there was a reduction in response in online of 9% (95% CI 3 to 14%; *p* = 0.003).

#### Patient age: under 75, 75 to 84 and older than 85 years old

There were 1049 people aged younger than 75, and the completion in the online group was 14% (38/270) versus 21% (55/267) in the face-to-face group. There were 594 people aged 75 to 84, and the completion was 4% (6/142) in the online group, compared to 23% (30/127) in the face-to-face group (*p* < 0.001). There were 401 people aged 85 or older, and 3% (3/103) responded in the online group and 4% (4/97) in the face-to-face group.

The results from the subgroup analysis did not indicate that patient age did not impact substantially on the study findings.

## Discussion

This randomised controlled trial assessed the use of a stroke-specific set of patient-facing outcome measures in 14 UK sites. In total, 2074 eligible people were selected using four different assessment methods: face-to-face, via the post, via the telephone and via online. The online assessment method was inferior and performed significantly worse than the other three assessment methods. With the exception of aphasia, where stroke survivors without aphasia were more likely to respond by post, this was a consistent association. The association with lower online completion was present regardless of age, stroke type (haemorrhage or infarct) and stroke severity.

The methods of administration of delivery were deliberately chosen to include face-to-face, telephone, online or postal assessment. All of these comparators are recommended by the Sentinel Stroke National Audit Programme (SSNAP) [6]. Currently, SSNAP requires every English and Welsh stroke survivor to be offered a 6-month follow-up appointment. The SSNAP audit suggests that this assessment can use any of these four methods of administration.

Why an online assessment should be inferior in this setting should be explored, particularly in terms of generalisability to non-stroke populations. This stroke cohort was predominantly an older population who, historically at least, is less fluent with online technologies. A recent Dutch study suggested that only 27% of frail older people who enrolled in a personal online health community used the system at least once a month [7]. It is possible that over time the older population will become far more IT aware and this method of delivery more usual. However, the subgroup analysis for all age groups showed stroke survivors failed to complete the online assessment, and an increased rate of uptake was not seen in the younger age group. This implies that stroke disease itself may not be a suitable condition in which to complete an electronic assessment. Stroke is a disabling condition, both physically and cognitively; hence, it is possible that stroke survivors do not have the ability, either physically or cognitively, to access the internet to respond. Hence, whether online assessments in other chronically disabling conditions, for example, rheumatoid arthritis, are warranted should also be explored. Our findings support the need for tailored support [8], in these types of chronic conditions. Another potential reason why online assessment performed poorly may be due to a comparatively simple follow-up method employed for online responses. Participants were sent a single URL address to type into a computer to access the 15 patient-facing questions. More sophisticated methods, such as text messaging direct links to online follow-up platforms, may engender a more active take-up. However, if the stroke population do not access the internet, this may be equally true of other modern technologies, such as using mobile phones. Finally, the assessment was undertaken at 6 months post stoke; therefore, it is possible that as stroke survivors continue to recover, then the use of online material may increase and this may become a more easily accessible format for this group. Conversely, with conditions that are likely to worsen over time, caution should be employed when using online assessments in patients with progressive symptoms, as an online method of delivery may be influencing the response rate rather than the condition which is being assessed.

It is less clear why stroke survivors without aphasia preferred to respond by post. One explanation would be that a postal response can be done at a patient’s own pace, unlike a telephone call or the need for a clinic visit. There was a suggestion that the affect may also have been driven by an increased postal response rate in those aged over 85 years, perhaps reflecting the increased challenges of clinic attendance or difficulty getting to the telephone, in the oldest old. Having impaired verbal communication (aphasia) would have led to the proposition that aphasic patients may respond more by post and less via the telephone, reflecting the verbal nature of the respond method. This may have been caused by random error due to a large amount of missing aphasia data, and this finding should be interpreted with caution.

Weaknesses need to be highlighted for this study. Firstly, the overall response rate for the study was low. While 23% of the study population consented to the study, ultimately only 18% returned the questionnaire. The low response rate may have been, at least in part, anticipated due to the nature of Zelen’s design. However, to our knowledge, there are no contemporary comparisons for an expected response rate of an online assessment in stroke, or indeed any other condition. The sample size calculation at the planning stage did not include a multiple testing adjustment and was increased during the study to reduce the risk of reporting a type II error. There are limited reports of what would be expected as a typical non-online response rate for a patient-reported outcome measure (PROM) in a stroke population, but the figure from this study (23%) does seem to be below these. For example, in an American Veterans study, assessing the Stroke Impact Scale, rates for postal and telephone responses were 45% and 69%, respectively [9]. In a study from the Australian Stroke Clinical Registry, which predominantly focused on cost, after two attempts at contact (which mirrors the findings in this study), the completion rates of telephone and postal follow-up were 73% and 68%, respectively [10]. A more comparable, recent UK study examining PROM collection by post in primary care demonstrated a response rate of 36.4% [11] for stroke survivors. This result was closer to the result demonstrated in this study and possibly more reflective, as the patients in this study were approached to take part following identification from a GP practice register, with an accompanying cover letter.

It could be anticipated that a response rate will be higher in a normal clinical setting where follow-up assessment would be requested by the clinical team as part of routine patient care. However, the low figure that was recorded in this study may reflect the disabling nature of stroke in general. Additionally, the 15 questions that were asked may have been too complicated for people with the residual stroke disability to complete, and further work should be done on the accessibility and ease of the use of all stroke focused PROMs.

Within medicine (and in general), the use of technology is ubiquitous and health care providers have an increased drive to use online assessment methods. This has perceived benefits in terms of ease of use for patients, automated data management for clinicians and financial benefits for health providers [12]. However, in this population, online follow-up was inferior to the current gold standard and, thus, not recommended. Further, online assessments will not replace some elements of longer-term health care follow-up, for example, medical examination or medication review. Policymakers and clinicians need to reflect on the use of online assessment for stroke survivors and similar other populations. Our findings may be due to older people lacking IT skills, or access to technology, and thus, these associations should be repeated to examine the changes over time.

## Conclusion

This randomised controlled trial approached over 2200 people throughout the UK. Of the four methods chosen, postal and the telephone were no worse than face-to-face contact. However, the online assessment method was inferior to face-to-face. It is possible that this is unique to stroke research. However, it suggests the introduction of online assessments needs further exploration before being routinely assumed to be globally beneficial for patients regardless of the underlying aetiology or age.

## Additional file

Additional file 1: Figure of the differences between methods of delivery. Key: **p < 0.01. **Figure S1.** The 15 questions assessed. **Figure S2.** Study consort diagram. Table S1. Displays the recruitment figures and participating sites. (DOCX 96 kb)

## Data Availability

The datasets used and/or analysed during the current study are available from the corresponding author on reasonable request.

## Abbreviations

aOR: Adjusted odds ratio
CI: Confidence interval
CNS: Clinical nurse specialist
GP: General practitioner
HRA: Health Research Authority
ICD: International Classification of Disease
KCTU: King’s College Trials Unit
NIHSS: National Institutes of Health Stroke Score
PIS: Participant information sheet
PROM: Patient-reported outcome measure
SD: Standard deviation
SOP: Standard operating procedures
SSNAP: Stroke Sentinel National Audit Programme
