# Supplementary material for: A multi-centre, UK-based, non-inferiority randomised controlled trial of 4 follow-up assessment methods in stroke survivors
Source: BMC Med. 2019 Jul 2;17:111. doi: 10.1186/s12916-019-1350-5 (PMC6604353; doi:10.1186/s12916-019-1350-5)
Supplement: Supplementary file 1 — Figure of the differences between methods of delivery. Key: **p < 0.01. Figure S1. The 15 questions assessed. Figure S2. Study consort diagram. Table S1. Displays the recruitment figures and participating sites. (DOCX 96 kb) [file 12916_2019_1350_MOESM1_ESM.docx]

Supplementary Data

Supplementary Figure 1: The 15 questions assessed

PROMIS Scale v1.2 – Global Health

22 August 2016 © 2010-2016 PROMIS Health Organization and PROMIS Cooperative Group Page 1 of 2

**Global Health**

| **Please respond to each question or statement by marking one box per row. Excellent** | | **Very**  good | | **Good** | | | **Fair** | | **Poor** | |
| --- | --- | --- | --- | --- | --- | --- | --- | --- | --- | --- |
| Global01 | In general, would you say your health is: ........... | | 🞎  5 | | 🞎  4 | 🞎  3 | | 🞎  2 | | 🞎  1 |
| Global02 | In general, would you say your quality of life is: .......................................................................... | | 🞎  5 | | 🞎  4 | 🞎  3 | | 🞎  2 | | 🞎  1 |
| Global03 | In general, how would you rate your physical health? ................................................................ | | 🞎  5 | | 🞎  4 | 🞎  3 | | 🞎  2 | | 🞎  1 |
| Global04 | In general, how would you rate your mental health, including your mood and your ability to think? ............................................................. | | 🞎  5 | | 🞎  4 | 🞎  3 | | 🞎  2 | | 🞎  1 |
| Global05 | In general, how would you rate your satisfaction with your social activities and relationships? ...................................................... | | 🞎  5 | | 🞎  4 | 🞎  3 | | 🞎  2 | | 🞎  1 |
| Global09r | In general, please rate how well you carry out your usual social activities and roles. (This includes activities at home, at work and in your community, and responsibilities as a parent, child, spouse, employee, friend, etc.) ...... | | 🞎  5 | | 🞎  4 | 🞎  3 | | 🞎  2 | | 🞎  1 |
| **Completely** | | **Mostly** | | **Moderately** | | | **A little** | | **Not at all** | |
| Global06 | To what extent are you able to carry out your everyday physical activities such as walking, climbing stairs, carrying groceries, or moving a chair? ................................................................ | | 🞎  5 | | 🞎  4 | 🞎  3 | | 🞎  2 | | 🞎  1 |

RiksStroke

Riksstroke, Medicincentrum, Norrlands universitetssjukhus

Are you able to walk?

Able to walk without help from another person with or without a device

Able to walk with help from another person

Unable to walk

Do you need help from anybody to go to the toilet?

I can manage going to the toilet without assistance

I need help to go to the toilet

Do you need help with dressing/undressing?

I can manage dressing/undressing without help

I need help dressing/undressing

International Consortium for Health Outcomes Measurement (ICHOM)

©ICHOM

Do you need a tube for feeding?

Yes

No

Do you have problems with communication or understanding?

Yes

No

Supplementary Table 1: Displays Recruitment Figures and participating sites

Supplementary Figure 2, Study Consort Diagram


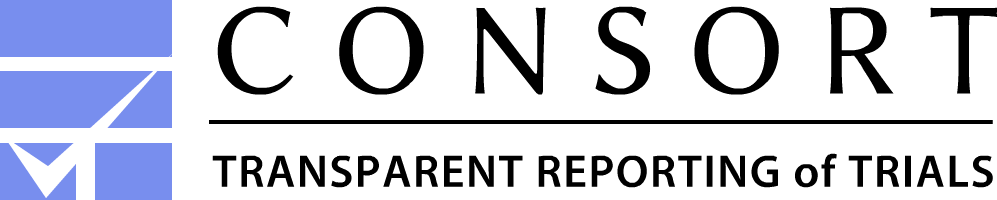


Assessment of Patient Reported Health Status Questions via Four Different Methods of Administration, in Stroke Survivors. A Randomised Controlled Trial.

A non-inferiority study of the response rate for four alternative methods of administration of 15 Patient Reported Health Status questions in stroke survivors.

IRAS ID 222226 CMPS ID 35499

**CONSORT 2010 Flow Diagram**

- Excluded (n= 143)

Met exclusion criteria (n=47)

Died prior to 6 month assessment being performed (n= 94)

Missing baseline Data (n=2)

Randomized (n= 2074)

Analysed Group Telephone (n= 513)
♦ Excluded from analysis (n=0)

Analysed Face 2 Face (n= 521)
♦ Excluded from analysis (n=0)

Analysed Group On Line (n= 515)
♦ Excluded from analysis (n=0)

Analysed Group Paostal (n= 525)
♦ Excluded from analysis (n=0)

Allocated to Group 3 (n= 513)

♦ Received allocated intervention (n= 513)

♦ Did not receive allocated intervention (n= 0 )

## Analysis

Allocated to Face 2 Face Method (n= 521)

♦ Received allocated intervention (n= 521)

♦ Did not receive allocated intervention (n= 0 )

Allocated to Group 1 (n= 515)

♦ Received allocated intervention (n= 515)

♦ Did not receive allocated intervention (n= 0 )

Allocated to Group 0 (n= 525)

♦ Received allocated intervention (n= 525)

♦ Did not receive allocated intervention (n= 0 )

## Allocation

## Enrollment

Assessed for eligibility (n= 2217)

## Follow-Up

Lost to follow-up (give reasons) (n=0)

Discontinued intervention (give reasons) (n= 0)

Lost to follow-up (give reasons) (n= 0)

Discontinued intervention (give reasons) (n= 0)
